# The Neuroelectromagnetic Inverse Problem and the Zero Dipole Localization Error

**DOI:** 10.1155/2009/659247

**Published:** 2009-06-17

**Authors:** Rolando Grave de Peralta, Olaf Hauk, Sara L. Gonzalez

**Affiliations:** ^1^Electrical Neuroimaging Group, Neurology Department, Geneva University Hospital, 24 Rue Micheli du Crest, 1211 Geneva 14, Switzerland; ^2^Neurodynamics Laboratory, Department of Psychiatry and Clinical Psychobiology, University of Barcelona, 08035 Barcelona, Catalonia, Spain; ^3^Cognition and Brain Sciences Unit, Medical Research Council, 15 Chaucer Road, Cambridge, CB2 7EF, UK

## Abstract

A tomography of neural sources could be constructed from EEG/MEG recordings once the neuroelectromagnetic inverse problem (NIP) is solved. Unfortunately the NIP lacks a unique solution and therefore additional constraints are needed to achieve uniqueness. Researchers are then confronted with the dilemma of choosing one solution on the basis of the advantages publicized by their authors. This study aims to help researchers to better guide their choices by clarifying what is hidden behind inverse solutions oversold by their apparently optimal properties to localize single sources. Here, we introduce an inverse solution (ANA) attaining perfect localization of single sources to illustrate how spurious sources emerge and destroy the reconstruction of simultaneously active sources. Although ANA is probably the simplest and robust alternative for data generated by a single dominant source plus noise, the main contribution of this manuscript is to show that zero localization error of single sources is a trivial and largely uninformative property unable to predict the performance of an inverse solution in presence of simultaneously active sources. We recommend as the most logical strategy for solving the NIP the incorporation of sound additional a priori information about neural generators that supplements the information contained in the data.

## 1. Introduction

Determining the neural origin and strength of sources producing scalp maps of electric or magnetic fields requires the solution of an inverse problem. This so-called neuroelectromagnetic inverse problem (NIP) lacks a unique solution. In spite of this serious difficulty, there is an active past and ongoing research on this field (see [1] for a recent review) because of the extreme clinical and research importance of the problem. A reliable optimal solution to the NIP is thus far the only possible alternative to study a direct reflection of neuronal activity in normal human subjects with the high temporal resolution required to trace the highly dynamic behavior of the human brain.

Several linear and nonlinear solutions based on a diversity of approaches have been proposed. However, independently of the approach used, we need to evaluate the reliability of the estimates provided by the inverse procedure selected. While there is interesting ongoing research on this topic [2–5], no definitive or general answer to this problem hitherto *exists*. One alternative to evaluate the localization features of linear inverse solutions is the so-called model resolution matrix (MRM) [6, 7], although the way to use it in the evaluations remains as a highly controversial point because of the following reasons. 

Some authors center their attention on the columns of the MRM, also called point spread functions (PSFs), that allow inferring how the solutions behave for single punctual sources. These authors consider the PSF as an adequate measure of the “goodness” of a linear inverse [8, 9]. An aspect to consider here is the existence in literature of two parallel definitions of the single (punctual) source localization error [10].

1. The bias in Dipole localization (BDL) defined in terms of the accuracy in estimating the location of each Cartesian component of the dipole. As such, it is a linear measure fully compliant with the linearity involved in the definition of the Model Resolution Matrix and can be directly estimated from the PSF.
2. The Dipole Localization Error (DLE) defined as the error attained in localizing the modulus of the current density vector. This definition conceptually disagrees with the use of MRM and PSF since the modulus is a nonlinear transformation of the individual dipole components not directly reflected by the PSF. Besides, linking the dipole localization error with the superposition principle is a blatant error since the basis of superposition is linearity. Although it certainly holds that the PSF of two simultaneously active dipoles is the sum of their individual PSFs, this is not the case for the DLEs. The widespread use of the dipole localization error concept obeys to historical and practical reasons since the modulus is the magnitude currently displayed in brain imaging.

All along this paper we will use the term single source to denote each of the three orthonormal (i.e., orthogonal with unitary norm) dipoles associated with a solution point. This is in agreement with the structure of the model resolution matrix where each solution point is represented by three columns. Consequently, each column corresponds to one and only one of three Cartesian components of a dipole. As typically used in this field, the term perfect localization will be used whenever the DLE or the BDL of a single source is zero independently of the off-diagonal elements of that column.

Two linear inverse solutions have been reported in the NIP literature to explicitly optimize the localization of single sources. The EPIFOCUS solution [11] aims to minimize DLE and BDL for both noisy and noiseless data for all sources in the solution space. In contrast, the sLORETA inverse solution [9] minimizes DLE and BDL only for noiseless data. 

Authors advocating the use of PSF employ the appealing argument of the superposition principle [12] as the basis to infer the capabilities of the solution for multiple source localization from results obtained on single source localization. They consequently concentrate their efforts in optimizing the columns of the MRM and will likely consider the zero dipole localization error as the ultimate goal to reach in the construction of inverse estimators. Another group of authors diverge from this point of view and insist that the performance of a linear inverse solution in the presence of multiple sources can only be inferred from the resolution kernels (the rows of the MRM). They consider the analysis of the PSF only valid for single source localization but not sufficient to describe the performance of distributed source models satisfactorily [3, 13]. They will therefore consider essential the incorporation of as much a priori information as possible into the solution to deal with the nonuniqueness, that is, they will aim to characterize the space where actual sources are contained [14, 15]. 

In this paper we introduce a “trivial” and easy-to-compute linear inverse solution coined Adjoint Normalized Approximation (ANA) that transforms the original inverse problem into a space in which the model resolution matrix shows optimal properties for single source localization. We demonstrate that in the transformed space, ANA inverse solution is able to correctly localize single sources in full extent, that is, with zero dipole localization bias and perfectly accurate strength. These properties are shown to be satisfied for arbitrary lead field models independently of the amount of scalp sensors. ANA solution is used to build a simple didactical example illustrating that perfect localization of single sources in position and strength *has* no implications for simultaneous source localization. The presented example serves to understand the emergence of spurious sources and how they totally distort the reconstruction when multiple sources are active. We further demonstrate that ANA can be applied to retrieve sources in the space of the original current density vector. Even if in this space the bias in dipole localization error is not zero everywhere, ANA solution is highly robust to noise outperforming the best methods presented so far for single source localization. Its robustness to noise and computational simplicity make of ANA a reasonable alternative for data generated by a single dominant source plus noise as can be the case in epilepsy. Still, ANA is more likely to contribute to further developments in this field, by providing the simplest possible evidence that optimizing single source localization is both trivial and useless. Therefore the only reasonable way to deal with the nonuniqueness of NIP is to add plausible physical and physiological constraints into the source space.

## 2. Methods

### 2.1. The Theoretical Basis of the Problem

The neuroelectromagnetic inverse problem (NIP), that is, the reconstruction of the current density vector inside the brain responsible for the electric and magnetic fields measured near/over the scalp, can be represented by a (first kind) Fredholm linear integral equation, denoting the relationship between the data measured at the external point, *d*(*s*), and the superposition of the contribution of the unknown current source density distribution at locations *r* inside the brain [16]:

(1) $$d\left(s\right)=\int_{\text{Brain}}^{}L\left(s,r\right)*j\left(r\right)dr.$$

The (vector) lead field function *L*(*s*, *r*) contains all the information about the boundary conditions as well as the media conductivities or permittivities for the electric and magnetic cases, respectively. The 3D vector *j*(*r*) denotes the unknown current density vector, and *r* is the 3D position variable running over the volume of the brain. 

Under experimental conditions, neither the measurements nor the lead field function is known for arbitrary surface/brain locations. However, assuming that the integral equation can be approximated by a discrete sum, (1) can be represented by an underdetermined system of linear equations:

(2) d=Lj.

Vectors **d** and **j** and matrix **L** represent the discretization of the continuous functions, that is, **d**_*k*_ = *d*(*s*_*k*_), **j**_*m*_ = *j*(*r*_*m*_), and **L**_km_ = *w*_km_ *L*(*s*_*k*_, *r*_*m*_), and *w*_km_ are the quadrature weights. 

All linear solutions of (2) can be obtained solving a variational problem [7]. This yields the inverse matrix **G** that, when applied to the measured data, produces the estimated current density vector, that is,

(3) $$\hat{j}=Gd.$$

Substitution of the measured data, as described in (2), into (3) yields the following fundamental equation for underdetermined linear systems:

(4) $$\hat{j}=Gd=GLj=Rj.$$

Here, **R** = **G** **L** denotes the model resolution matrix (MRM) describing the relationship between the estimate and the original magnitudes of the current density. In simpler terms, (4) tells us that our estimates separate from the original values by the transformation **R**. The nearer this matrix is to the identity matrix, the better the estimated solution resembles the original sources. 

For the noisy case where **d** = **L** **j** + **N** **o** **i** **s** **e**, we can always rewrite it as **d** = **L** **j** + **L** **j**_*n*_ where **j**_*n*_ is the minimum norm solution of the equation **N** **o** **i** **s** **e** = **L** **j**_*n*_, and thus

(5) $$\hat{j}=Gd=GL\left(j+j_{n}\right)=R\left(j+j_{n}\right).$$

For the particular example discussed here, the unknown current density vector contains the three Cartesian components at each solution point. Correspondingly, each solution point will be represented by 3 columns and 3 rows of the MRM. The rows of **R** are known as the resolution kernels [17]. Each resolution kernel provides information on how simultaneously active sources affect the estimates of **j** at the component associated to the row. The columns of **R** are the point spread functions (PSFs) and reflect the quality of single source reconstruction. That is, each column corresponds to the current source density estimated by the inverse solution when the associated unitary single source is active alone. Based on the linearity of matrix products, to compute the current source estimated for simultaneously active sources it is enough to add the associated columns. For further details about how to compute the bias in dipole localization and the dipole localization errors from the PSF, see [7, 10].

### 2.2. The Adjoint Normalized Approximation (ANA) of the Inverse

It is evident that for every invertible matrix **W**, the following change of variable can be applied to (2):

(6) $$d=LW^{-1}Wj=ℒZ,$$

where *ℒ* = **L** **W** ^−1^ and **Z** = **W** **j**. Let us define **W** as the diagonal matrix containing the norm of each column of **L**. It follows from the definition of **W** that it is a diagonal square matrix and thus invertible. Therefore (6) is identical to the original problem formulation in (2); what has been done is a simple change of variable where the model matrix is the column normalized lead field, and the unknown is the variable **Z.**

To obtain a unique solution to (6) in the space of the transformed variable **Z**, we need to invert the model matrix *ℒ*. Since we are dealing with an underdetermined inverse problem, matrix *ℒ* is noninvertible. A typical choice for inverse problems is to use the Moore-Penrose pseudoinverse. We rather propose to use a particularly simple approximation of the inverse of a matrix, the adjoint or transpose (not to be confused with the adjugate matrix composed by the cofactors). This simple choice satisfies the third and fourth Moore-Penrose conditions, while violating the first two [18], that is, if **A** is a matrix (or vector) and **G** is its generalized inverse, then it must hold that (1) **A** **G** **A** = **A**. (2) **G** **A** **G** = **G**. (3) (**A** **G**)^*t*^ = **A** **G**, and (4) (**G** **A**)^*t*^ = **G** **A**. It also follows that the pseudoinverse of **G** is **A**. Therefore the proposed Adjoint Normalized Approximation (ANA) inverse is given by

(7) $$G={ℒ}^{t}={\left(LW^{-1}\right)}^{t}=W^{-1}L^{t}.$$

There is a close relationship between ANA and EPIFOCUS. While EPIFOCUS computes the pseudoinverse of three lead field columns (i.e., three single sources) associated with one solution point, ANA corresponds to the computation of the pseudoinverse of each column (i.e., single source) separately. This is straightforward since the Moore-Penrose inverse of a normalized (unitary norm) vector is the transposed vector which fulfills all the four conditions of the pseudoinverse mentioned before. We would also note that the adjoint corresponds to the simpler initial approximation of the inverse for iterative processes. The normalized adjoint is a step forward fulfilling one property of the inverse, that is, the product with the original matrix yields one at the diagonal. As it was the case for EPIFOCUS [11], the simulations of the next section confirm that ANA properties are not a consequence of the weighting or the transposition alone but a combined effect.

## 3. Results

### 3.1. Theoretical Properties of ANA's Resolution Matrix

According to (4), the resolution matrix associated with the transformed variable **z** is given by

(8) $$R={ℒ}^{t}ℒ=W^{-1}L^{t}LW^{-1}.$$

From this, it follows that the resolution matrix of ANA inverse solution is the product of the transposed normalized lead field times the normalized lead field. Therefore the resolution matrix is symmetric.

Further properties of the resolution matrix **R** (8) can be derived by noting that the elements of the *i*th column of **R** are given by the scalar product of the potential map produced by the *i*th source with the potential map of all other sources. This derives from the fact that each column of **L** represents the electric potential or magnetic field pattern measured at/near the head surface when only the *i*th dipolar source is active with unitary strength (“forward solutions”). Since each dipole produces a different activation map, it is then clear that each pair of columns of **L** is noncollinear. The resolution matrix of ANA in the transformed space *ℒ* necessarily inherits the property of noncollinearity from **L** since the only change is a normalization factor. Consequently, the *i*th column of **R** contains the correlation coefficients between the *i*th potential pattern and the potential patterns of all other sources. Since the correlation coefficient between a given potential map with itself is necessarily one, then the elements at the main diagonal of **R** (the map autocorrelations) are inevitably equal to one. The nondiagonal elements, representing the correlations between one given map and all other maps, are necessarily lower than one since different unitary dipoles are unable to produce identical scalp maps. Since these properties hold for all sources, that is, all columns of **R**, then, the maximum of each column, defining the bias in dipole localization, is reached at the main diagonal and is exactly one. Thus, the following properties hold for the resolution matrix of this inverse independently of the lead field model considered.

1. The point-spread functions (columns of **R**) reach their maxima at the diagonal elements trivially leading to perfect reconstruction of the positions of all single sources (all Cartesian components of the dipole at each solution point).
2. Because the diagonal of the resolution matrix is one (due to normalization), the intensity of the estimated source is exactly the intensity activity of the original source.
3. Since **R** is symmetric, then the resolution kernels shapes are close to the ideals attaining the maximum value at the correct places.

### 3.2. Does Perfect Localization of Single Sources Imply Correct Localization of Multiple Active Sources?

The ideal properties of ANA's resolution matrix described in the previous section are independent from the lead field model. This implies that they will hold even for arbitrarily small sensor configurations and very large solution spaces provided that there are no collinear columns in the lead field. We have exploited this fact to construct a simple numerical example that might help to shed light on several aspects influencing the behavior of linear inverse solutions in the presence of multiple active sources. The computational simplicity of ANA will facilitate the task to readers interested in further simulating its behavior with simultaneous sources. 

**Figure 1:**
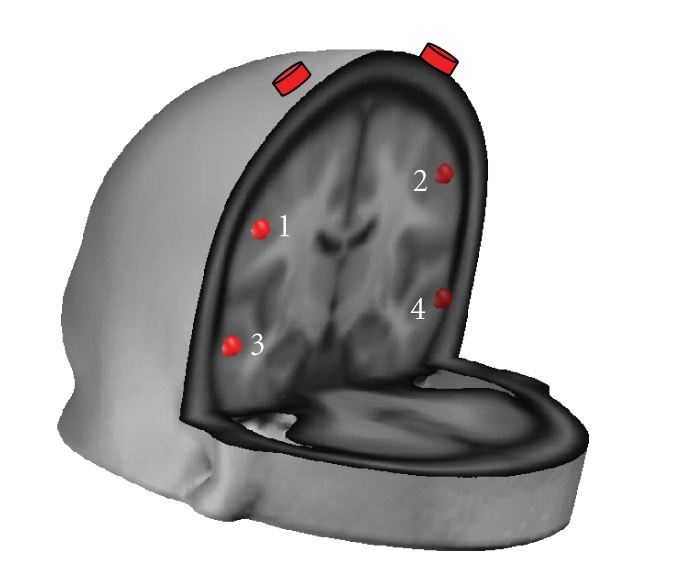
*Electrodes and solution points used for the analysis of ANA resolution matrix**. ***The two electrodes are located at the approximated positions of C3 and C4.

The example given here considers the case of two EEG sensors and four solution points as depicted in Figure 1. The four solution points lie in a coronal plane below the arc at which the two sensors are placed. Sensors are placed at the approximate positions of electrodes C3 and C4 of the international 10/20 placement system. The lead field was computed using a semirealistic head model derived from the Montreal Neurological Institute (MNI) average brain using the SYSMAC procedure described in [19]. It is noteworthy that the selection of the lead field matrix parameters (conductivities, electrode positions, and solution points) will have little effect on the main results described below. This argument justifies our selection of a very small problem to allow portraying the full model resolution matrix and its subsequent understanding.

In the case of this simple example, the current density vector is a 12 component vector of the form

(9) $$j=\left[\begin{array}{cccc} \begin{array}{ccc} j_{x}^{1} & j_{y}^{1} & j_{z}^{1} \end{array} & \begin{array}{ccc} j_{x}^{2} & j_{y}^{2} & j_{z}^{2} \end{array} & \begin{array}{ccc} j_{x}^{3} & j_{y}^{3} & j_{z}^{3} \end{array} & \begin{array}{ccc} j_{x}^{4} & j_{y}^{4} & j_{z}^{4} \end{array} \end{array}\right].$$

This vector is formed by the three Cartesian components of the dipoles (subscripts **x**, **y**, **z**) linked to each solution point (superscripts 1, 2, 3, 4). The spatial distribution of the modulus of the current density vector can be computed using

(10) $$j^{i}=\sqrt{{\left(j_{x}^{i}\right)}^{2}+{\left(j_{y}^{i}\right)}^{2}+{\left(j_{z}^{i}\right)}^{2}}\;\text{for}\;\;i=1,2,3,4,$$

resulting in the vector of the modulus given by

(11) $$j_{m}=\left[\begin{array}{cccc} j^{1} & j^{2} & j^{3} & j^{4} \end{array}\right].$$

Table 1 shows the model resolution matrix **R** associated with ANA inverse solution for this problem. This is a 12 × 12 matrix where each group of three rows (or columns) represents the resolution kernels (or impulse responses) linked to the three Cartesian components of a dipole at the corresponding solution point. 

The theoretical properties derived in the previous section obviously hold for the problem presented. The main diagonal is filled by ones that are the dominant elements within their respective rows (and columns since the matrix is symmetric). A first aspect to note is that while the recovery of each Cartesian component of the dipole (if alone) is perfect, the recovery of the modulus is not. Perfect recovery of the modulus can be obtained with ANA inverse by stating the original problem for the modulus rather than for the individual dipolar components. This can be done by determining a priori the orientation as in SAM beamformer [20] or by reformulating the problem as proposed in [21]. Here we stick, for the sake of simplicity and compliance with the MRM linearity, to the case of the component-by-component estimation.

The following two simple examples illustrate how the model resolution matrix is used to derive the inverse solution estimates for a single active source and for two simultaneously active sources. 

According to (4), if the “true” current density vector has the form (9), then the ANA inverse solution estimate is given by the product of the MRM and the “true” vector. Let us imagine that the true source distribution is formed by a single active source, which is the *z*-component of the first solution point with strength *k*. In this case, the true vector is according to (9) given by [0,0, **k**, 0, 0, 0, 0, 0, 0, 0, 0, 0]. The current density vector estimated by ANA is the product of *R* by this column vector that yields precisely the third column of *R* multiplied by *k*. Therefore, ANA solution leads to a maximum at the third component of the first point (third element of the third column), and the estimated strength is exactly *k*. Note that all the other elements in the reconstruction, although smaller than the third one, are different from zero. All the nonzero elements are spurious sources.

In the same way, the reconstruction of each single active source of unitary strength is given by the PSF (column of MRM) linked to this source component. While the maximum always occurs at the right position and the source strength is correctly estimated, the reconstruction is rather noisy and contains spurious activity (ghost sources). This spurious activity appears at sites where the true source strength is zero and is a consequence of nonzero off-diagonal elements of the resolution matrix. To better understand the origin of nonzero off-diagonal elements in the MRM, we should remember that its *i*th column contains the correlation coefficients between the *i*th potential pattern and the potential patterns of all other sources. Nonzero off-diagonal elements of the resolution matrix appear therefore at the position of sources leading to correlated scalp patterns. For the particular case of ANA inverse solution, the value at the off-diagonal elements will be identical to the correlation coefficient between the respective potential patterns. Different sources might produce highly similar scalp potential patterns (highly correlated patterns) inducing large off-diagonal elements and therefore spurious sources. 

Not only will off-diagonal elements lead to noisy single source reconstruction but also, even worse, they will totally mislead multiple source reconstruction. To see how, let us return to our example of Table 1 and assume that sources 1 and 12 are active (both with unitary strength). In practical terms, this means that the *x*-component of a dipole is active at the first solution point and the *z*-component of a dipole is active at the fourth solution point. The reconstruction provided in this case will be equal to the sum of columns 1 and 12 of the resolution matrix, and its numerical values are given in Table 2. 

The largest positive value of the reconstruction appears at source component number four and therefore at the second solution point. The largest absolute value appears at the source component number six which also belongs to the second solution point. The modulus of the vector, given in Table 3, shows similar results. This means that neither the component-by-component reconstruction nor the modulus shows maxima at the actual source locations at solution points one and four. In fact the fourth solution point has the smallest modulus, and its active component the third smallest estimated strength. The failure of the solution to retrieve the two simultaneously active sources is once again due to the existence of large off-diagonal elements in the MRM. Hopefully, this numerical example helps to understand that the naïve intuitive application of the superposition principle to this problem is erroneous since exclusively based on the diagonal elements of the MRM. 

As for a comparison, we depict on Table 4 the resolution matrix for the Minimum Norm (i.e., Moore Penrose pseudo inverse) solution. Note that while it is symmetric, the maxima for each row (or column) are not necessarily at the main diagonal. Note also that several elements are zero for the numerical precision (3 decimal digits) used. 

### 3.3. Single Source Localization with ANA in the Original Source Space of *j* and Synthetic Noisy Data

We have shown so far that ANA solution is capable to provide perfect localization of single sources within the space of the transformed variable **Z**. However, it is clear that on the original source space the symmetry of the resolution matrix will not hold and that we cannot insure that MRM elements are bounded. However, based on the rationale behind ANA and EPIFOCUS, there is no reason to believe that this will prevent ANA to correctly localize single sources in the original source space. To shed some light on this issue, we can resort to simulations with single sources. This issue is of concern because the problem of single dipole localization under the assumption of a dominant generator remains of interest in several practical neurophysiological applications such as epilepsy [22–25]. Linear inverse solutions constitute an appealing alternative to nonlinear dipole localizations because of their higher computational simplicity and their possibilities to be applied to irregular solution spaces required for modeling patient's brains [11]. We might therefore wonder if the good features of ANA for single source localization hold within the original source space **j**. For practical applications in clinical and research routine, we expect a solution which guarantees accurate localization but which is also robust, that is, capable to deal with experimental noise and modeling errors (sensor location, approximate head conductivities, etc.) and particularly with changes in the pattern/map of the dominant source induced by other weaker sources that are simultaneously active.

In this section we present some simulation results to study how much the theoretical performance degrades with noise in the original source space **j**. We compare the localization results for four linear solutions including three that are highly efficient for single source localization: (1) ANA, (2) EPIFOCUS [11, 26], and (3) sLORETA [9]. The fourth solution, that is, (4) the Moore-Penrose inverse of the normalized lead field was also included to confirm that the results of ANA are not simply due to the weighting strategy introduced in its design. 

For reproducibility and compatibility with previous publications, we use in this section a lead field model corresponding to the sensor configuration and solution space described in ISBET NEWSLETTER number 6, December 1995, Grave and Gonzalez, 2000, Grave et al. 2001. Namely, a unit radius 3-shell spherical head model (Ary et al., 1981), with solution points confined to a maximum radius of 0.8. The sensor configuration comprises 148 electrodes. The solution space consists of 817 points on a regular grid with an intergrid distance of 0.133 cm, corresponding to 2451 focal sources. To simulate noisy data, we added to each electrode uncorrelated random noise in the range ±15% of the amplitude of the noiseless data. DLE and BDL are divided by the size of the grid unit (0.133) and are evaluated for *x* values in the set [0, 0.5, 1, 1.5, 2, 2.5, 3, 3.5, 4, 4.5, 5, 5.5, 6, 6.5, 7]. For each value *x*_*i*_, we compute

1. the empirical Probability Distribution Function, defined as follows: Probability(*x*_*i*_) = {Number of sources with errors ≦ *x*_*i*_}/2451;
2. the empirical density function defined for *x*_*i*_ < 7 as follows: Density(*x*_*i*_) ={Number of sources with errors in [*x*_*i*_, *x*_*i*+1_]}/2451.

Note that while the empirical density function describes the performance for each eccentricity range, the probability function provides a global assessment about how fast the maximum asymptotic value is attained.

**Figure 2:**
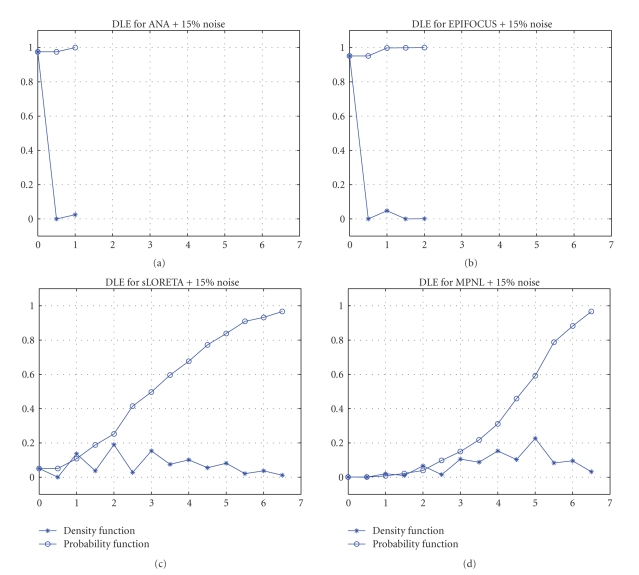
*Dipole Localization Error (DLE) results with synthetic data with 15*% *noise*. The model is composed of 148 electrodes and 2451 single dipoles at 817 solution points. Probability and Density functions (vertical axis) are plotted versus error sizes (horizontal axis) measured in grid units. Despite the noise in the data, DLE for EPIFOCUS and ANA are never bigger than two grid units while sLORETA and MPNL errors can be higher than 6 grid units.

Figure 2  presents the dipole localization error for ANA, EPIFOCUS, sLORETA, and MPNL inverse in the localization of the 2451 single sources when the data is contaminated with 15% of noise. While the results for sLORETA and MPNL are equally erratic for noisy data (Figure 2), they clearly differ for noiseless data (not shown here) where sLORETA attains zero DLE whereas MPNL remains unreliable. In contrast ANA and EPIFOCUS have very similar behavior for noiseless (not shown) and noisy data (Figure 2). All regularization parameters tested for sLORETA (namely, *λ* = 0, 1e-6, 0.1, 1, 10) yield similar erratic results for noisy data. Figure 2  depicts the results for sLORETA for just one of the values tested (*λ* = 0). 

**Figure 3:**
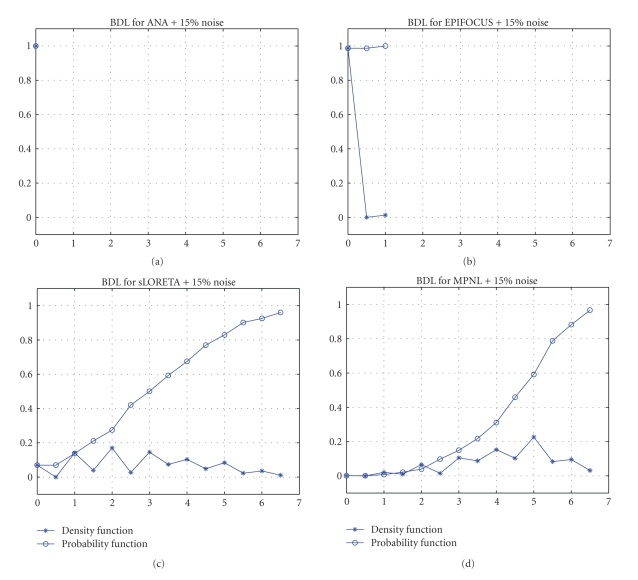
*Bias in dipole localization results for noisy data with 15*% *noise*. Model includes 148 electrodes and 2451 single dipoles placed at 817 solution points. Probability and Density functions (vertical axis) are plotted versus error sizes (horizontal axis). Despite the noise in the data, BDL for EPIFOCUS and ANA are never bigger than two grid units while sLORETA and MPNL errors can be higher than 6 grid units.

Figure 3  presents the bias in dipole localization for ANA, EPIFOCUS, sLORETA, and MPNL inverse in the localization of the 2451 single sources when the data is contaminated with 15% of noise. For the noiseless (not shown) data sLORETA and ANA attain zero BDL for all the sources, while for the noisy data (Figure 3) only ANA remains at zero BDL followed by EPIFOCUS. MPNL and sLORETA produce errors as large as 6.5 grid units. All regularization parameters tested for sLORETA (namely, *λ* = 0, 1e-6, 0.1, 1, 10) yield similar erratic results. The results shown in Figure 3  for sLORETA correspond to a regularization parameter of *λ* = 1.

## 4. Discussion

The ANA inverse solution described in this paper is, to the best of our knowledge, the first linear solution to the NIP simultaneously fulfilling (in the transformed space) the three following properties: (1) symmetric resolution matrix; (2) perfect single source localization, and (3) perfect estimation of single source strength. Probably this is also the simplest (in the sense of numerical complexity) solution with these properties. Importantly, such properties stem from the theoretical resolution matrix and therefore hold for arbitrary (with noncollinear columns) lead field models. 

In case we accept that perfect single source localization, that is, correct estimation of the location and the source strength as in ANA or correct estimation of the location as in sLORETA, suffices to insure perfect multiple source reconstruction, we must conclude that ANA or sLORETA is the solution to the NIP. This statement is in flagrant contradiction to any rationale. The mistake resides in the assumption that perfect single source localization, defined as zero DLE or zero BDL, implies accurate multiple source localization. This implication is true only for the ideal resolution matrix with zero off-diagonal elements, which is impossible for an underdetermined problem. As demonstrated here, ANA solution is theoretically perfect for single source reconstruction but failed in the simplest case of two simultaneously active sources. As shown in the example, the reason for such failure is the existence of nonzero off-diagonal elements within the model resolution matrix that are ignored by the DLE or BDL. As we saw, nonzero off-diagonal elements appear as a consequence of the correlation between scalp potential (magnetic fields) patterns associated with different punctual sources. Such off-diagonal elements are inherent to the problem statement (the lead field model) and will appear for all linear inverse solutions (e.g., sLORETA, MPNL, EPIFOCUS, etc.), although to different extent. Note that while noiseless data imply the selection of a single MRM column, noisy data can be interpreted as an additional source (generating the noise) implying that multiple columns of the MRM should be added to get the final current density estimator. As shown before, off-diagonal elements might dominate such reconstruction even in the noiseless case. However, as long as the components of the additional source are lower than the correlation between patterns of neighboring dipoles, ANA (and the closely related EPIFOCUS) should yield low BDLs. Simulations suggest that this is not the case for sLORETA or MPNL with errors up to 6.5 grid units. 

Importantly, it is widely accepted that localization accuracy will indefinitely improve by increasing the number of scalp recording sensors. While increasing the number of sensors augments the amount of information about the underlying sources, it does also enhance the correlation (redundancy) between the rows of the lead field matrix, that is, the way that one sensor sees all the sources. The increase in correlation between rows results in unstable (sensitive to noise) problems that need special regularization strategies to avoid noise amplification. This trade-off between the independent information conveyed by the new measurements and their redundancy will define a practical superior bound to the amount of electrodes to be used for source localization purposes. 

We have seen that neither the perfect single source localization nor the unlimited increase in the amount of recording sensors will definitively solve the NIP. Obviously, the only remaining choice is to incorporate as much a priori information as possible about the generators into the problem. Such information should be independent of the information already contained in the measurements. A priori information can be incorporated within the discrete formalism by a right-side transformation of the lead field matrix, which in turn can be interpreted as a change of variable. Only this procedure, illustrated here for ANA solution (see (6)), will allow to effectively modify the shapes of resolution kernels. Nevertheless the question remains open which of these right-side modifications of the lead field will result in correctly centered resolution kernels. Examples of right-side transformations of the lead field already employed in the NIP literature are the irrotational source model of ELECTRA [15, 27] or the transformed lead field based on predefined directions of the sources used in SAM [8, 20].

The value of ANA solution is not only didactical. As shown by our simulation results, ANA can be applied to retrieve sources in the space of the original variable **j**. Although in this space the dipole localization error is not zero everywhere, the bias in dipole localization remains zero and the results are very robust to noise. In this sense ANA solution compares to the more robust methods presented so far. Its computational simplicity, easiness of application to irregularly distributed solution spaces, and localization capabilities make of ANA a reasonable alternative for the analysis of data generated by a single dominant source plus noise. Such assumptions are not rare in one of the most important clinical applications of source localization, namely, the determination of the site of onset of epileptic activity [22, 25].

It is worth mentioning that the limitations described here are not specific to linear inverse solutions, and they will certainly appear under a different mask for nonlinear inverse procedures. While these difficulties are easily analyzed within the linear framework because of the possibilities offered by the model resolution matrix formalism, they actually reflect the ill-posed nature of the original inverse problem. Therefore, unless useful a priori information is found that cannot be incorporated within linear inverses, we see no good reasons to replace the comfortable linear framework with its inherent computational and interpretational simplicity. 

The evaluation and design of linear inverse solutions over last decade have been misguided by the idea that only solutions able to accurately localize a large proportion of single sources will succeed in the quest for constructing a tomography of neural generators [9, 12]. Hopefully, the examples and arguments in this paper will help to reorient research within this field to the characterization of properties of neural generators as the sole way to overcome the nonuniqueness of the NIP inverse problem. Research in this direction is not doomed to failure, and existing inverse solutions can lead to relevant and novel findings within neuroscience when correctly exploited and interpreted. While often overlooked, some of the limitations of linear inverse solutions to the NIP are shared by the fMRI. For instance, the absolute size of the fMRI contrast signal cannot be relied upon to measure the amplitude of the neural responses at two different cortical locations [28]. In a similar manner we should be cautious comparing current source density estimates at two different solution points since amplitude estimates vary as a function of the actual current distribution as well as the diagonal and off-diagonal elements of the MRM. However, we can rely either on experimental contrasts as done with fMRI or on measures invariant to scale transformations such as spectral measures derived from temporal information of the estimated sources [29–31] to improve the reliability of the information retrieved from the inverses.

## 5. Conclusions

Here we introduced a linear inverse solution coined ANA which fulfills several optimal properties for the localization of single sources. We demonstrated by means of the model resolution matrix formalism that ANA localizes correctly the location and the amplitudes of all single sources. These properties hold for arbitrary lead fields and for arbitrarily small sensor configurations. This fact was exploited to introduce simple examples that clarify how spurious sources are formed and their large relevance for simultaneous source reconstruction. We further showed that ANA solution is highly robust to noise, outperforming established methods for single source localization (sLORETA and EPIFOCUS). Its robustness to noise and computational simplicity make ANA a reasonable alternative for data generated by a single dominant source plus noise, as can be the case in epilepsy. 

The most important contribution of this manuscript is to provide definitive evidence that the apparently reasonable (although naïve) idea of inferring the behavior of linear solutions from their single source localization properties proves false. It is thus concluded that zero localization error alone is a trivial and useless property unable to predict the performance of an inverse solution in presence of simultaneously active sources. We expect that these results will help researchers to guide their choices of inverse methods, in methods development as well as for clinical and neuroscientific applications. We also hope that it will stimulate further interest in finding neurophysiologically plausible constraints that can be used as a priori information in the NIP, which should be the ultimate goal in this endeavour.

## Figures and Tables

**Table 1:** *The* *resolution matrix for ANA and the configuration presented in Figure 1.* The 12-by 12-model resolution matrix for the configuration of Figure 1 is composed by two electrodes and 4 solutions points. The 12-dimensional unknown current density vector (9) is composed by the 3 Cartesian components of the dipolar moment for each solution point.

1	0.48	0.94	0.48	−0.84	−0.75	−0.67	−0.86	−0.93	−0.10	−0.93	0.16
0.48	1	0.74	0.99	−0.87	−0.94	−0.97	−0.85	−0.13	0.81	−0.14	0.94
0.94	0.74	1	0.75	−0.97	−0.92	−0.88	−0.98	−0.75	0.23	−0.76	0.48
0.48	0.99	0.75	1	−0.87	−0.94	−0.97	−0.85	−0.14	0.81	−0.15	0.94
−0.84	−0.87	−0.97	−0.87	1	0.98	0.96	0.99	0.60	−0.43	0.61	−0.66
−0.75	−0.94	−0.92	−0.94	0.98	1	0.99	0.98	0.46	−0.57	0.47	−0.77
−0.67	−0.97	−0.88	−0.97	0.96	0.99	1	0.95	0.36	−0.66	0.37	−0.83
−0.86	−0.85	−0.98	−0.85	0.99	0.98	0.95	1	0.62	−0.40	0.63	−0.63
−0.93	−0.13	−0.75	−0.14	0.60	0.46	0.36	0.62	1	0.45	0.99	0.20
−0.10	0.81	0.23	0.81	−0.43	−0.57	−0.66	−0.40	0.45	1	0.44	0.96
−0.93	−0.14	−0.76	−0.15	0.61	0.47	0.37	0.63	0.99	0.44	1	0.19
0.16	0.94	0.48	0.94	−0.66	−0.77	−0.83	−0.63	0.20	0.96	0.19	1

**Table 2:** *The reconstruction provided by ANA when multiple sources are active is erroneous despite the perfect reconstruction of both sources alone*. Current density vector reconstruction for EEG data generated when the first and the last single sources are simultaneously active with unitary amplitude.

1.16	1.42	1.42	1.43	−1.50	−1.52	−1.51	−1.50	−0.72	0.86	−0.74	1.16

**Table 3:** *Modulus of the current density vector of Table 2*. Each value corresponds to the strength of the source at each solution point as computed using (10).

2.32	2.57	2.25	1.62

**Table 4:** *Resolution matrix for the minimum norm solution and the configuration presented in Figure 1.* Even though it is symmetric, the maxima are not always located at the main diagonal.

0.00	−0.02	−0.02	0.00	0.00	−0.01	0.00	0.00	0.00	0.00	0.00	0.00
−0.02	0.5	0.45	0.00	0.15	0.02	0.00	0.09	0.01	0.00	0.09	0.11
−0.02	0.45	0.45	−0.01	−0.01	0.16	0.00	0.06	0.05	0.00	0.06	0.11
0.00	0.00	−0.01	0.00	0.02	−0.02	0.00	0.00	−0.01	0.00	0.00	0.00
0.00	0.15	−0.01	0.02	0.5	−0.44	0.00	0.08	−0.13	0.00	0.08	−0.01
−0.01	0.02	0.16	−0.02	−0.44	0.45	0.00	−0.05	0.13	0.00	−0.06	0.04
0.00	0.00	0.00	0.00	0.00	0.00	0.00	0.00	0.00	0.00	0.00	0.00
0.00	0.09	0.06	0	0.08	−0.05	0.00	0.02	−0.02	0.00	0.02	0.01
0.00	0.01	0.05	−0.01	−0.13	0.13	0.00	−0.02	0.04	0.00	−0.02	0.01
0.00	0.00	0.00	0.00	0.00	0.00	0.00	0.00	0.00	0.00	0.00	0.00
0.00	0.09	0.06	0.00	0.08	−0.06	0.00	0.02	−0.02	0.00	0.02	0.01
0.00	0.11	0.11	0.00	−0.01	0.04	0.00	0.01	0.01	0.00	0.01	0.03
